# Association of Cytotoxic T-Lymphocyte Antigen 4 (*CTLA4*) and Thyroglobulin (*TG*) Genetic Variants with Autoimmune Hypothyroidism

**DOI:** 10.1371/journal.pone.0149441

**Published:** 2016-03-10

**Authors:** Hinal Patel, Mohmmad Shoab Mansuri, Mala Singh, Rasheedunnisa Begum, Minal Shastri, Ambikanandan Misra

**Affiliations:** 1 Pharmacy Department, Faculty of Technology & Engineering, The Maharaja Sayajirao University of Baroda, Vadodara, Gujarat, India; 2 Biochemistry Department, Faculty of Science, The Maharaja Sayajirao University of Baroda, Vadodara, Gujarat, India; 3 Medicine Department, Faculty of Medicine, Sir Sayajirao Gaekwad Hospital, The Maharaja Sayajirao University of Baroda, Vadodara, Gujarat, India; Beijing University of Chemical Technology, CHINA

## Abstract

Autoimmune hypothyroidism is known to be caused by immune responses related to the thyroid gland and its immunological feature includes presence of autoimmune antibodies. Therefore the aim was to analyze presence of anti-TPO antibodies in hypothyroidism patients in Gujarat. Cytotoxic T-Lymphocyte Antigen 4 (*CTLA4*) is one of the susceptibility genes for various autoimmune diseases. Hence, exon1 +49A/G and 3’UTR CT60A/G single nucleotide polymorphisms (SNPs) in *CTLA4* and its mRNA expression levels were investigated in autoimmune hypothyroidism patients. Thyroglobulin (*TG*) is known to be associated with autoimmune thyroid disorders and thus exon 33 (E33) SNP in *TG* was investigated. We analyzed the presence of anti-TPO antibodies in the plasma samples of 84 hypothyroidism patients and 62 controls by ELISA. PCR-RFLP technique was used for genotyping of polymorphisms. s*CTLA4* and fl*CTLA4* mRNA expression levels were assessed by real time PCR. 59.52% of hypothyroid patients had anti-TPO antibodies in their circulation. The genotype and allele frequencies differed significantly for +49A/G *(p* = 0.0004 for +49AG, *p* = 0.0019 for +49GG & *p* = 0.0004 for allele), CT60 (*p =* 0.0110 for CT60AG, *p =* 0.0005 for CT60GG & *p*<0.0001 for allele) and *TG* E33 (*p* = 0.0003 for E33TC *p*<0.0001 for E33CC& *p*<0.0001 for allele) SNPs between patients and controls. Patients had significantly decreased mRNA levels of both s*CTLA4* (*p* = 0.0017) and fl*CTLA4* (*p*<0.0001) compared to controls. +49A/G and CT60 polymorphisms of *CTLA4* were in moderate linkage disequilibrium. Logistic regression analysis indicated significant association of CT49A/G, CT60A/G and *TG* exon 33 polymorphisms with susceptibility to autoimmune hypothyroidism when adjusted for age and gender. Our results suggest +49A/G and CT60 polymorphism of *CTLA4* and E33 polymorphism of *TG* may be genetic risk factors for autoimmune hypothyroidism susceptibility and down regulation of both forms of *CTLA4* advocates the crucial role of *CTLA4* in pathogenesis of autoimmune hypothyroidism.

## Introduction

Hypothyroidism is an endocrine disorder characterized by decreased activity of thyroid gland leading to insufficient production of thyroid hormones. Subclinical or asymptomatic hypothyroidism is characterized by elevated thyrotropin level and normal serum thyroid hormones level. Whereas, there remains elevated thyrotropin but decreased thyroid hormones serum levels in case of overt or clinical hypothyroidism [1] [2].

In India, hypothyroidism used to usually be categorized under the iodine deficient disorders and represented based on total goiter rate. Government of India has adopted the universal salt iodization program and since then there has been a decline in goiter prevalence in various parts of the country [3–7]. As per World health organization (WHO) assessment report India has undergone transition from iodine deficient state to iodine sufficient state [8–10]. A large, cross-sectional, comprehensive study recently carried out in adult population across the country, indicates about 10.9% prevalence of hypothyroidism [11]; whereas, the prevalence of hypothyroidism in the developed countries is about 4–5% [12, 13]. This indicates even though most of the regions of India have been made iodine sufficient there is still high prevalence of hypothyroidism. Hence, underlying pathogenesis may involve a complex interplay of genetic, environmental and endogenous factors and not only iodine deficiency. Clinical investigation of patients in India does not include evaluation of thyroid autoantibodies and hence iodine deficiency is believed to be the sole candidate for hypothyroidism pathogenesis which may not be the case.

Autoimmune hypothyroidism is characterized by gradual destruction of the thyroid gland due to loss of thyroid cells, leading to thyroid hormone deficiency. The immunological features of this disorder include the presence of anti-thyroidperoxidase (anti-TPO) antibodies and, less commonly, anti-thyroglobulin (anti-TG) antibodies, abnormalities in the circulating T cell population and a goiter with lymphocytic infiltration [14, 15]. To date, significant progress has been made in identifying and characterizing genes involved in the disease pathogenesis. As both environmental and genetic factors appear to play a role in disease susceptibility [16], the precise mechanism for the pathogenesis of this disorder is not fully understood. The cytotoxic T-lymphocyte antigen 4 (*CTLA4*) and thyroglobulin (*TG*) genes have been considered to be major genetic factors involved in the development of autoimmune hypothyroidism.

The *CTLA4* gene on human chromosome 2q33 is one of the candidate genetic markers for autoimmune diseases, encodes a cell surface molecule that is expressed on the surface of activated T lymphocytes and has the most remarkable function of down regulation of the immune response [17]. *CTLA4* binds to the ligands, B7-1 and B7-2, as CD28 but with a 20–50-fold higher affinity. The interaction between *CTLA4* and B7 plays an essential role in regulation of self-tolerance, and hence susceptibility to autoimmune diseases [18]. The *CTLA4* gene produces two different *CTLA4* protein isoforms: full length *CTLA4* (fl*CTLA4*) and soluble *CTLA4* (s*CTLA4*). The fl*CTLA4* serves as a transmembrane receptor on activated T cells to inhibit cell proliferation. The role of s*CTLA4* is not yet known but it has been suggested that s*CTLA4* can block the B7-CD28 interaction by acting as functional receptor for B7 antigens and thus can interfere with the co-stimulation signal and inhibit T-cell proliferation [19].

Several polymorphic sites in the *CTLA4* gene such as promoter -318 C/T[20], exon 1 +49 A/G [21, 22], microsatellite (AT)n repeat in the 3’-untranslated region (UTR) of exon [23] and three single nucleotide polymorphisms (SNPs) in the 6.1-kb 3’ non-coding region such as CT60, JO31 and JO30 have been reported to be associated with the organ-specific autoimmune disorders in several racial groups.[18, 24][25, 26] Among them, 3’ UTR CT60, -318 C/T and exon 1 +49 A/G SNPs are the highly polymorphic markers associated with autoimmune endocrinopathies [27]. According to prior studies on the association the *CTLA4* gene with the development of various autoimmune diseases, the exon 1 +49 A/G polymorphism in *CTLA4* exon 1 has been reported to be involved in the development of autoimmune diseases including Graves’ disease [28] and Hashimoto thyroiditis [27]. The CT60 in the 3’UTR region of *CTLA4* gene is also the most promising locus for the autoimmune thyroid diseases [29]. The meta-analysis study shows consistent associations of Graves’ disease and Hashimoto thyroiditis with CT60 [30] and clarifies the important role of the *CTLA4* locus in determining the risk of autoimmune thyroid diseases.

The *TG* gene on 8q24 locus has been strongly linked with autoimmune thyroid diseases (AITD). Previous studies have demonstrated that an exon 10–12 SNP cluster and an exon 33 (E33) SNP are significantly associated with autoimmune thyroid diseases because amino acid substitution that occurs due to this polymorphism predisposes to autoimmune thyroid diseases [31].

Hypothyroidism diagnosis is limited to determination of thyroid hormone and thyrotropin serum levels and evaluation of autoimmune antibodies in patients is not currently practiced in India. We therefore investigated the presence of anti-TPO antibodies in patients with hypothyroidism and also analyzed the frequencies of *CTLA4* 3’ UTR CT60, exon 1 +49 A/G and *TG* E33 polymorphisms and *CTLA4* expression in autoimmune hypothyroidism patients and controls from Gujarat as indicators of thyroid disorder susceptibility.

## Subjects and Methods

### Subjects

The study plan was approved by ‘Human Scientific and Ethics Review Committee for Human Research’, Faculty of Medicine, The Maharaja Sayajirao University of Baroda, Vadodara, Gujarat, India. The importance of the study was explained to all participants and written consent was obtained from all patients and controls. The study group included 84 hypothyroidism patients comprised of 78 females and 6 males who referred to S.S.G. Hospital, Vadodara. The diagnosis of hypothyroidism was based on thyroid profile analysis (serum T_3_, T_4_ and TSH levels) and patients had no other associated autoimmune diseases. A total of 62 ethnically and sex-matched unaffected individuals were included as controls in this study. The control group comprised 55 females and 7 males (S1 Table). None of the healthy individuals had any evidence of hypothyroidism and any other diseases. The study plan was approved by the Institutional ethics committee for human research (IECHR), Faculty of Medicine, The Maharaja Sayajirao University of Baroda, Vadodara, Gujarat, India.

### Estimation of anti-Thyroid Peroxidase (anti-TPO) antibodies levels

In the present study, plasma from hypothyroidism patients was examined to find the levels of anti-TPO antibodies compared to controls. Plasma samples of 84 hypothyroidism patients and 62 controls were analyzed for the presence of anti-TPO antibodies by ELISA. 5 ml venous blood was collected from the patients and healthy subjects in K_3_EDTA coated vacutainers (BD, Franklin Lakes, NJ 07417, USA) and plasma was extracted. Presence of anti-TPO antibodies were assessed by ELISA method as per manufacturer’s protocol (Genway Biotech, Inc. San Diego, CA). Absorbance of all wells was measured at 450nm using 620nm as reference wavelength.

### Determination of s*CTLA4*, fl*CTLA4* and *GAPDH* mRNA expression

#### RNA extraction and cDNA synthesis

Total RNA from whole blood was isolated and purified using RibopureTM- blood Kit (Ambion inc. Texas, USA) following the manufacturer’s protocol. RNA integrity was verified by agarose gel electrophoresis/ ethidium bromide staining and O.D. 260/280 absorbance ratio >1.95. RNA was treated with DNase I (Ambion inc. Texas, USA) before cDNA synthesis to avoid DNA contamination. One microgram of total RNA was used to prepare cDNA. cDNA synthesis was performed using the Verso cDNA Synthesis Kit (Thermo scientific, Lithuania, EU) according to the manufacturer’s instructions using Mastercycler Gradient PCR (Eppendorf, Germany).

#### Real-time PCR

The levels of full length, soluble *CTLA4* and *GAPDH* transcripts were measured by real-time PCR using gene specific primers (S2 Table) (Eurofins, Bangalore, India). Expression of GAPDH gene was used as a reference. Real-time PCR was performed in duplicates in 20 μl volume using LightCycler®480 SYBR Green I Master (Roche Diagnostics GmbH, Mannheim, Germany) following the manufacturer’s instructions and carried out in the Light Cycler 480 Real-Time PCR (Roche Diagnostics GmbH, Mannheim, Germany). The thermal cycling conditions included an initial activation step at 95°C for 10 min, followed by 45 cycles of denaturation, annealing and amplification. The fluorescent data collection was performed during the extension step. At the end of the amplification phase a melt curve analysis was carried out on the product formed. The value of Cp was determined by the first cycle number at which fluorescence was greater than the set threshold value.

### Genotyping of *CTLA4* gene exon 1 +49A/G and 3’ UTR CT60A/G polymorphisms and *TG* gene exon 33 polymorphism

Genomic DNA was extracted from whole blood using ‘QIAamp DNA Blood Kit’ (QIAGEN Inc., Valencia, CA 91355, USA) according to the manufacturer’s instructions. Polymerase chain reaction–restriction fragment length polymorphism (PCR-RFLP) was used to genotype exon 1 +49A/G and 3’ UTR CT60A/G polymorphisms of *CTLA4* gene and E33 polymorphism of *TG* gene and amplification was performed using Mastercycler Gradient PCR (Eppendorf, Germany). according to the protocol: 95°C for 10 minutes followed by 30 cycles of 95°C for 30 seconds, primer (S2 Table) dependent annealing for 30 seconds, and 72°C for 30 seconds. The amplified products were checked by electrophoresis on a 2.0% agarose gel stained with ethidium bromide.

Restriction enzymes (New England Biolabs, Beverly, MA) used for digesting amplicons of exon 1 +49A/G and 3’ UTR CT60A/G of *CTLA4* gene and E33 polymorphism of *TG* gene are given in S2 Table. 15 μL of the amplified products were digested for 16 hours at 37°C with 5 U of the corresponding restriction enzyme. The digestion products with 100/50 base pair DNA ladder (Bioron, Ludwigshafen am Rhein, Germany) were resolved in 3.5% agarose gels stained with ethidium bromide and visualized under UV transilluminator.

### Statistical analysis

Evaluation of the Hardy-Weinberg equilibrium (HWE) was performed for the polymorphisms in patients and controls by comparing the observed and expected frequencies of the genotypes using chi-square analysis. The distribution of the genotypes and allele frequencies of *CTLA4* exon 1 +49A/G and 3’ UTR CT60A/G and *TG* E33 polymorphisms for patients and control subjects were compared using the chi-square test using Prism 5 software (Graphpad software Inc; San Diego CA, USA, 2007). Logistic regression analysis was applied to evaluate whether *CTLA4* exon 1 +49A/G and 3’ UTR CT60A/G and *TG* E33 polymorphisms predict the susceptibility to autoimmune hypothyroidism when adjusted for age and gender by using by SPSS statistics 23.0 (IBM SPSS Inc., Chicago, IL). Haplotype analysis was carried out using http://analysis.bio-x.cn/myAnalysis.php.[32] The linkage disequilibrium (LD) coefficients D’ = D/Dmax and r^2^-values for the pair of the most common alleles at each site were estimated using the Haploview programe version 4.1.[33] Differences were considered as statistically significant if the *p*-value was less than 0.025 due to Bonferroni’s correction for multiple testing of +49A/G and CT60A/G SNPs in *CTLA4* whereas, differences were considered to be statistically significant if the *p*-value was less than 0.05 for *TG* SNP. Odds ratio (OR) with respective confidence interval (95% CI) for disease susceptibility was also calculated. Relative expression of both fl*CTLA4* and s*CTLA4* in patient and control groups was plotted and analyzed by nonparametric unpaired t-test using Prism 5 software (Graphpad software Inc; San Diego CA, USA, 2007). The statistical power of detection of the association with the disease at the 0.05 level of significance was determined by using the G* Power software [34].

## Results

### Anti-TPO antibody levels in hypothyroidism patients and controls

It was found that 59.52% (n = 50) of hypothyroidism patients (n = 84) had anti-TPO antibodies in their blood circulation suggesting that autoimmunity may play an important role in the pathogenesis of the disease moreover, these autoimmune hypothyroidism patients had significantly increased anti-TPO antibody levels as compared to controls (*p*<0.0001).

### The expression of fl*CTLA4* and s*CTLA4* transcripts

**Fig 1:**
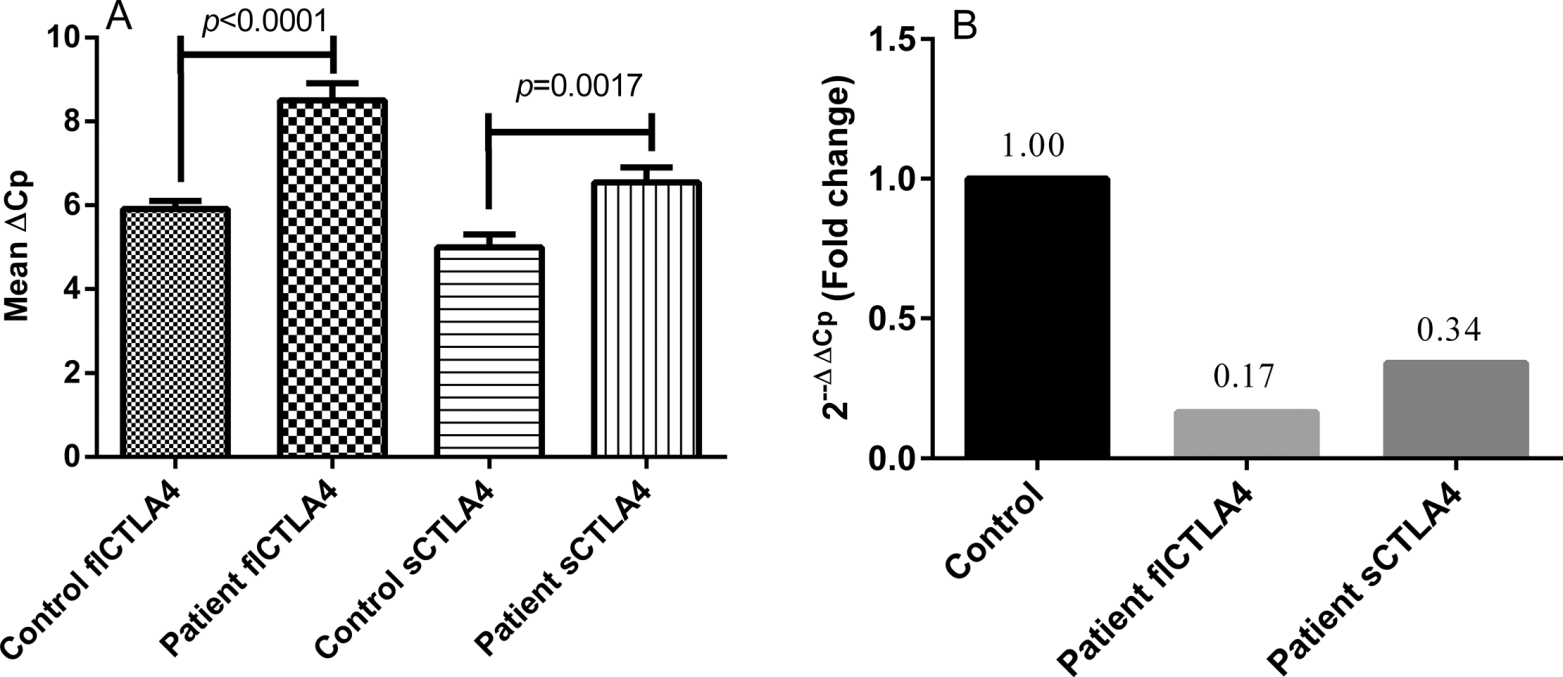
**Relative gene expression of fl*CTLA4* and s*CTLA4* in controls and autoimmune hypothyroidism patients: (A)** Expression of fl*CTLA4* and s*CTLA4* mRNA in 45 autoimmune hypothyroidism patients and 60 controls as suggested by Mean ΔCp. Autoimmune hypothyroidism patients showed significantly reduced mRNA levels of fl*CTLA4* (*p*<0.0001) and s*CTLA4* (*p* = 0.001) as compared to controls. **(B)** Expression fold change of fl*CTLA4* and s*CTLA4* in 45 autoimmune hypothyroidism patients and 60 controls showed approximately 0.166 and 0.342 fold decrease as determined by 2^-ΔΔCp^ method respectively.

Comparison of the findings showed significantly decreased expression of both full length and soluble *CTLA4* in autoimmune hypothyroidism patients than in controls after normalization with *GAPDH* expression (*p*< 0.0001 and *p* = 0.01 respectively) (Fig 1A). The 2^-ΔΔCp^ analysis showed 0.166 and 0.342 fold decrease in the expression of fl*CTLA4* and s*CTLA4* mRNA expression, respectively, in patients as compared to controls (Fig 1B).

### Genotype-phenotype correlations for fl*CTLA4* and s*CTLA4* in autoimmune hypothyroidism patients and controls

**Fig 2:**
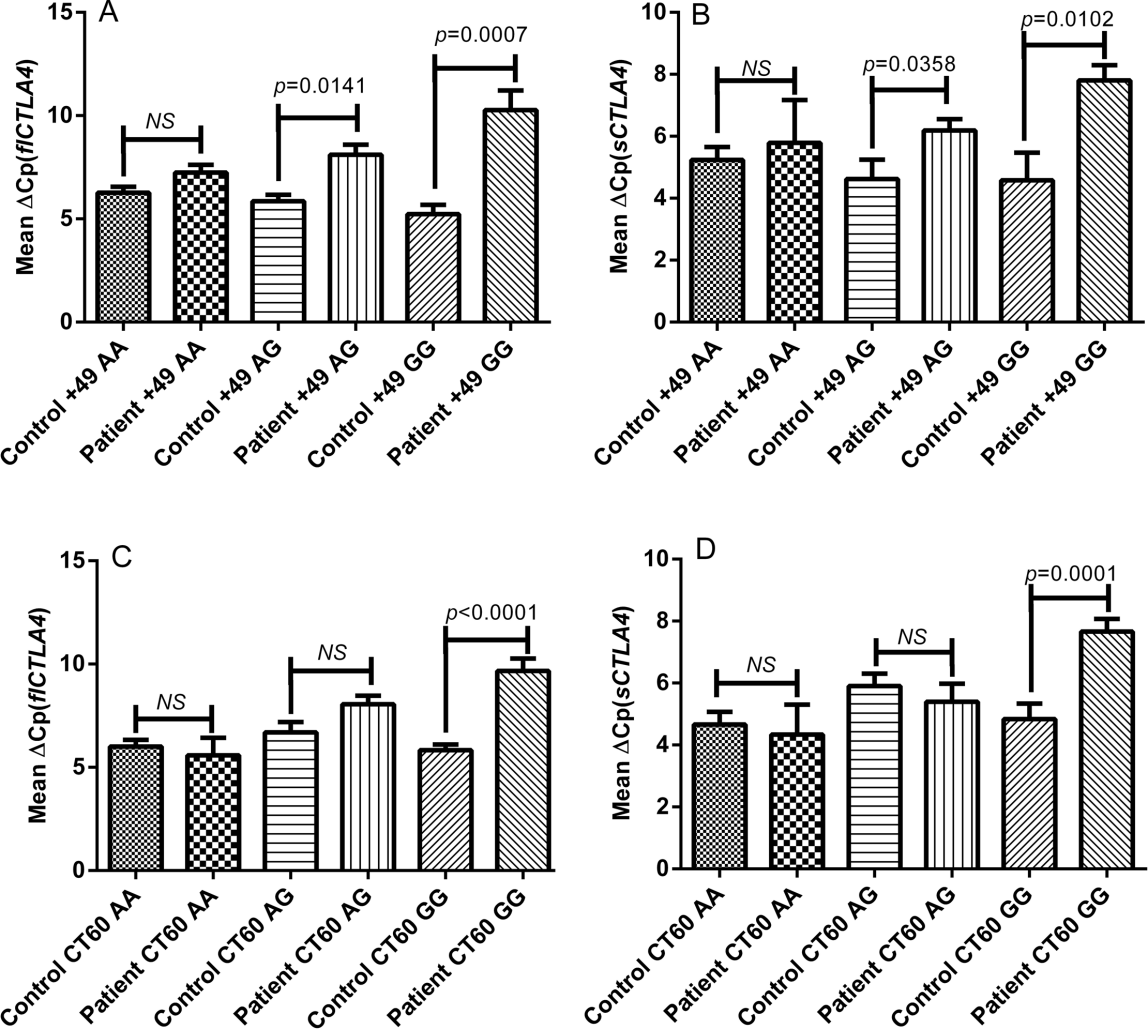
**Genotype—phenotype correlation of exon 1 +49A/G and 3’ UTR CT60A/G polymorphisms of fl*CTLA4* and s*CTLA4* in controls and autoimmune hypothyroidism patients: (A)** Relative mRNA expression of fl*CTLA4* with respect to +49A/G genotypes in 45 patients and 60 controls. None of the three genotypes AA (*p* = 0.0667), AG (*p* = 0.0141) and GG (*p* = 0.0007) in patients showed significant difference for fl*CTLA4* expression as compared to controls a suggested by Mean ΔCp. [*NS* = non-significant] **(B)** Relative mRNA expression of s*CTLA4* with respect to +49A/G genotypes in 45 patients and 60 controls. None of the three genotypes AA (*p* = 0.6260), AG (*p* = 0.0358) and GG (*p* = 0.0102) in patients showed significant difference for fl*CTLA4* expression as compared to controls a suggested by Mean ΔCp. [*NS* = non-significant] **(C)** Relative mRNA expression of fl*CTLA4* with respect to CT60A/G genotypes in 45 patients and 60 controls. GG genotype showed significant decrease in the levels of fl*CTLA4* mRNA (*p*<0.0001) in patients as compared to AA and AG genotype (*p* = 0.5817 & *p* = 0.2379 respectively) as suggested by Mean ΔCp. [*NS* = non-significant] **(D)** Relative mRNA expression of s*CTLA4* with respect to CT60A/G genotypes in 45 patients and 60 controls. GG genotype showed significant decrease in levels of s*CTLA4* mRNA (*p* = 0.0001) in patients as compared to AA and AG genotype (*p* = 0.7099 & *p* = 0.7478 respectively) as suggested by Mean ΔCp. [*NS* = non-significant].

Analysis of the mRNA expression of fl*CTLA4* and s*CTLA4* based on the +49A/G and CT60A/G genotypes of 45 autoimmune hypothyroidism patients and 60 controls was performed. The expression levels of fl*CTLA4* and s*CTLA4* for AA genotypes of exon 1 +49A/G polymorphism did not differ significantly in autoimmune hypothyroidism patients as compared to controls (*p* = 0.0667 and *p* = 0.6260 respectively) (Fig 2A & 2B). However, the expression levels of both fl*CTLA4* and s*CTLA4* were decreased significantly for AG (*p* = 0.0141 and *p* = 0.0358 respectively) and GG (*p* = 0.0007 and *p* = 0.0102 respectively) genotype of exon 1 +49A/G polymorphism in autoimmune hypothyroidism patients as compared to controls (Fig 2A & 2B). The expression levels of fl*CTLA4* and s*CTLA4* for AA (*p* = 0.5817 and *p* = 0.7099 respectively) and AG (*p* = 0.2379 and *p* = 0.7478 respectively) genotypes of CT60 polymorphism did not differ significantly in autoimmune hypothyroidism patients as compared to controls (Fig 2C & 2D). However, the expression levels of both fl*CTLA4* and s*CTLA4* were decreased significantly for GG genotype of CT60 polymorphism in autoimmune hypothyroidism patients as compared to controls (*p*<0.0001 and *p* = 0.0001 respectively) (Fig 2C & 2D).

The expression levels of fl*CTLA4* were found to be significantly decreased and associated with GG and AG haplotypes in patients and controls (*p* = 0.0091 and *p* = 0.0429 respectively). Other two haplotypes, such as, AA and GA did not differ with respect to fl*CTLA4* expression levels in patients and controls (*p* = 0.1271 & *p* = 0.3191). However, s*CTLA4* expression levels were found to be significantly decreased and associated with GG haplotypes in patients and controls (*p* = 0.0411); whereas, other three haplotypes: AA, AG and GA did not differ with respect to s*CTLA4* expression levels in patients and controls (*p* = 0.8129 & *p* = 0.1948 & *p* = 0.2925respectively).

### Ratio of s*CTLA4* and fl*CTLA4* mRNA expression in autoimmune hypothyroidism patients and controls

**Fig 3:**
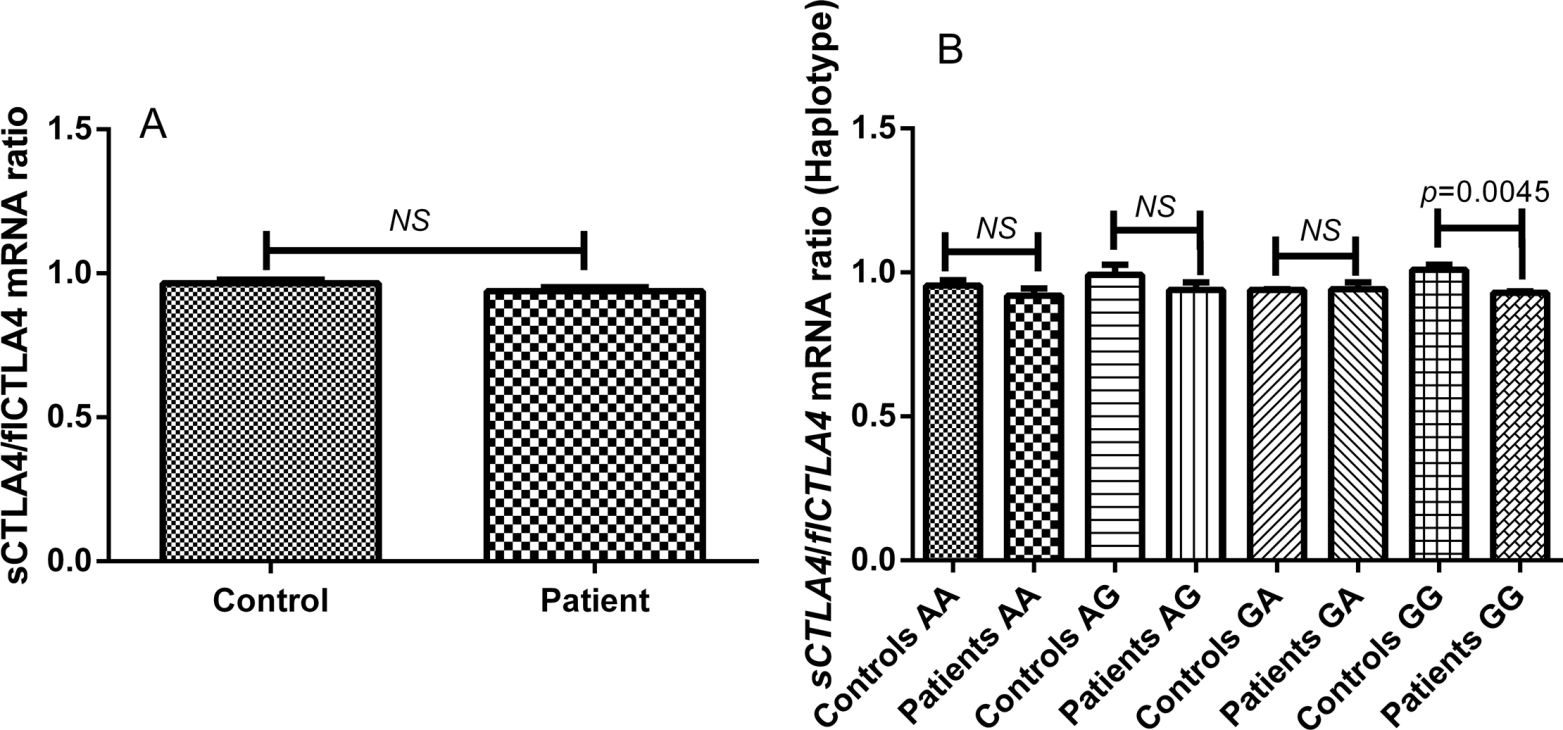
**Ratio of s*CTLA4* and fl*CTLA4* mRNA expression in controls and autoimmune hypothyroidism patients: (A)** s*CTLA4*/fl*CTLA4* mRNA ratio was measured in 45 autoimmune hypothyroidism patients and 60 controls which was not found altered (*p* = 0.1360). [*NS* = non-significant] **(B)** s*CTLA4*/fl*CTLA4* mRNA ratio was analyzed with respect to (+49A/G: CT60A/G) haplotypes in patients and controls. AA, AG and GA haplotypes did not show significant difference in the s*CTLA4*/fl*CTLA4* mRNA ratio (*p* = 0.4165, *p* = 0.2964 and *p* = 0.9481), however, GG haplotype showed significant increase in the s*CTLA4*/fl*CTLA4* mRNA ratio in patients compared to controls (*p* = 0.0045 respectively). [*NS* = non-significant].

The expression level of s*CTLA4* and fl*CTLA4* was also analyzed as ratio of s*CTLA4*: fl*CTLA4* in autoimmune hypothyroidism patients and controls. There was no significant difference in the ratio of s*CTLA4* to fl*CTLA4* mRNA expression between patients and controls (*p* = 0.1360) (Fig 3A).

**Fig 4:**
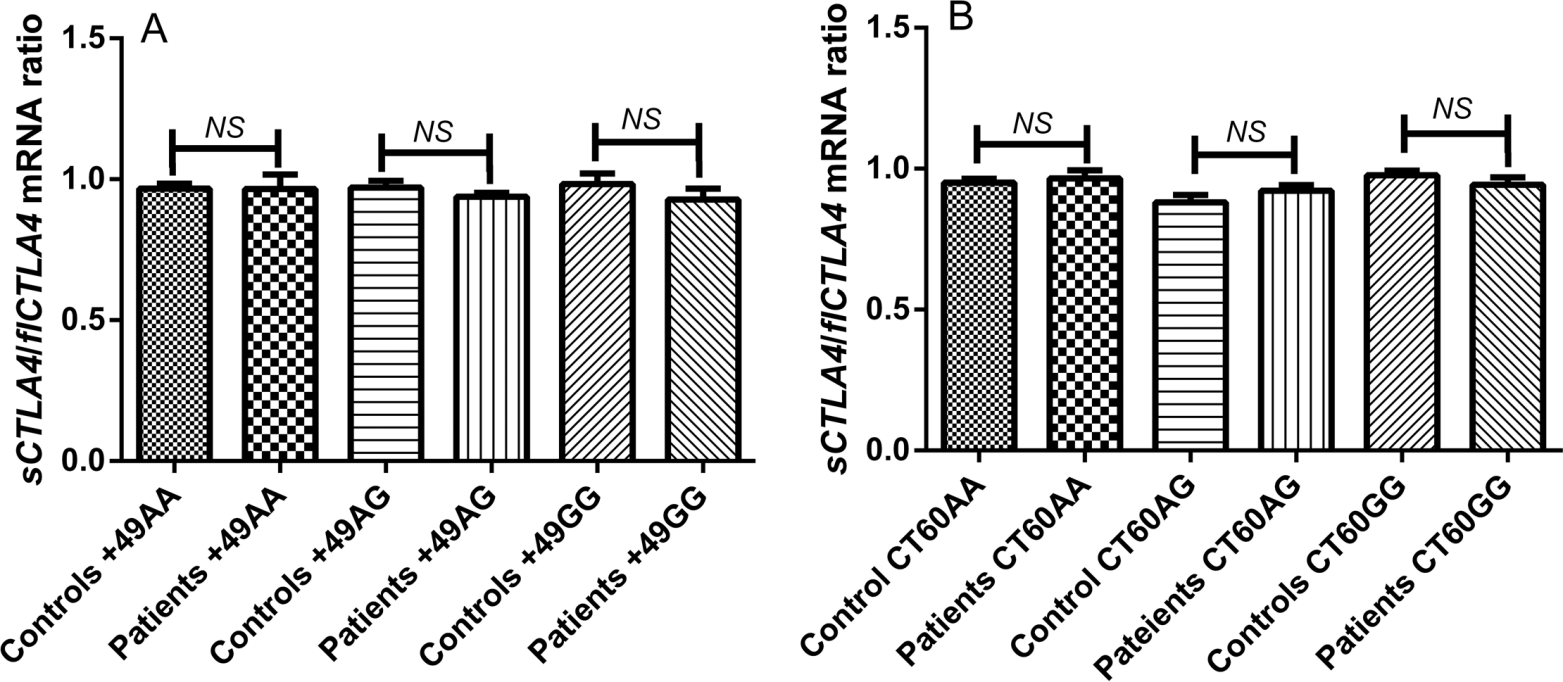
**Ratio of s*CTLA4* and fl*CTLA4* mRNA expression with respect to +49A/G and CT60A/G genotypes in controls and patients: (A)** s*CTLA4*/fl*CTLA4* mRNA ratio was analyzed with respect to +49A/G genotypes in patients and controls. None of AA, AG and GG genotypes showed significant difference in the s*CTLA4*/fl*CTLA4* mRNA ratio in patients compared to controls (*p* = 0.9724, *p* = 0.2378 and *p* = 0.3405 respectively). [*NS* = non-significant] **(B)** s*CTLA4*/fl*CTLA4* mRNA ratio was analyzed with respect to CT60A/G genotypes in patients and controls. None of AA, AG and GG genotypes showed significant difference in the s*CTLA4*/fl*CTLA4* mRNA ratio in patients compared to controls (*p* = 0.6213, *p* = 0.4425 and *p* = 0.2940 respectively). [*NS* = non-significant].

None of AA, AG and GG genotypes of exon 1 +49A/G polymorphism show any significant difference for the ratio of s*CTLA4* and fl*CTLA4* mRNA expression in patients compared to controls (*p* = 0.9724, *p* = 0.2378 and *p* = 0.3405). Similarly, the ratio of s*CTLA4* and fl*CTLA4* mRNA expression was not found to significantly differ for AA, AG and GG genotypes of CT60 polymorphism in patients compared to control (*p* = 0.6213, *p* = 0.4425 and *p* = 0.2940 respectively) (Fig 4B).

Moreover, ratio of s*CTLA4* and fl*CTLA4* mRNA expression did not differ significantly for the haplotypes AA, AG and GA in patients and controls (*p* = 0.159, *p* = 0.068 and *p* = 0.966 respectively) (Fig 3B). But patients with GG haplotype showed significant increase in the ratio of *sCTLA4* to *flCTLA4* mRNA expression compared to controls (*p* = 0.0045).

### Analysis of association between *CTLA4* gene exon 1 +49A/G polymorphism and susceptibility to autoimmune hypothyroidism

PCR-RFLP for +49A/G polymorphism yielded a 271 bp undigested product corresponding to G allele and 249 bp and 22 bp digested products corresponding to A allele. The three genotypes identified by 3.5% agarose gel electrophoresis were: AA homozygous, AG heterozygous and GG homozygous for +49A/G polymorphism of *CTLA4* gene (S1A Fig).

AG and GG genotypes of Exon 1 +49A/G polymorphism of *CTLA4* gene when compared with AA genotype between patients and control by chi-square test-2x2 contingency table showed to increase susceptibility to autoimmune hypothyroidism (*p* = 0.0004 and odds ratio 5.333; *p* = 0.0019 and odds ratio 5.779 respectively) (Table 1). Furthermore logistic regression analysis showed that, when adjusted for age and gender, AG and GG genotype of Exon 1 +49A/G polymorphism of *CTLA4* gene increase the risk of autoimmune hypothyroidism by 4.319 fold (95% CI: 1.408 to 13.249, *p* = 0.011) and 4.309 fold (95% CI: 1.102 to 16.855, *p* = 0.036) respectively (Table 2). From logistic regression, age and gender of the patients were not found to be associated with susceptibility to autoimmune hypothyroidism as age and gender matched controls were selected in this study (Table 2). However our data (S1 Table) suggest that autoimmune hypothyroidism is common in females than in males which is in accordance with previous prevalence studies on autoimmune thyroid disorders [35]. The mean age of onset is 38 years for females whereas for males it cannot be concluded because of relatively small sample size (S1 Table).

**Table 1:** Association studies for *CTLA4* gene exon 1 +49A/G and 3’UTR CT60A/G polymorphisms and *TG* gene exon 33 polymorphism in autoimmune hypothyroidism patients.

SNP	Genotype or allele	Patients (Frequency) n = 49	Controls (Frequency) n = 62	*P* for Association	Odds ratio (95% CI)
*CTLA4* Exon1 (+49A/G)	AA	8(0.16)	32(0.52)	R	1
	AG	28(0.57)	21(0.34)	0.0004^a^	5.333(2.043 to 13.92)
	GG	13(0.27)	9(0.14)	0.0019^b^	5.778(1.829 to 18.25)
	A	44(0.45)	85(0.69)	0.0004^c^	
	G	54(0.55)	39(0.31)		
*CTLA4* (CT60A/G) 3’UTR	AA	4(0.08)	22(0.35)	R	1
	AG	15(0.31)	17(0.27)	0.0110^a^	4.853(1.360 to 17.31)
	GG	30(0.61)	23(0.37)	0.0005^b^	7.174(2.169 to 23.73)
	A	23(0.23)	61(0.49)	<0.0001^c^	
	G	75(0.77)	63(0.51)		
*TG* Exon33	TT	4(0.08)	29(0.47)	R	1
	TC	28(0.57)	27(0.43)	0.0003^a^	7.519(2.329 to 24.27)
	CC	17(0.35)	6(0.10)	< 0.0001^b^	20.54(5.065 to 83.30)
	T	36(0.37)	85(0.69)	<0.0001^c^	
	C	62(0.63)	39(0.31)		

‘n’ represents number of Patients/ Controls, CI refers to Confidence Interval, R refers to Reference Group

^a^ and ^b^ represents Patients vs. Controls (genotype) using chi-squared test with 2 × 2 contingency table for AA vs AG and AA vs GG of +49A/G and CT60, and respectively for TT vs CT and TT vs CC of *TG* exon33.

^c^ represents Patients vs. Controls (allele) using chi-squared test with 2 × 2 contingency table.

**Table 2:** Association of *CTLA4* exon1 +49A/G, 3’ UTR CT60A/G and *TG* exon33 with autoimmune hypothyroidism when adjusted for age and gender using logistic regression.

SNP		*p* value	Odds ratio (95% CI)
*CTLA4* Exon1 (+49A/G)	AA(26)	R	1
	AG(32)	0.011	4.319 (1.408 to 13.249)
	GG(53)	0.036	4.309 (1.102 to 16.855)
*CTLA4* (CT60A/G) 3’UTR	AA(40)	R	1
	AG(49)	0.009	7.096 (1.616 to 31.171)
	GG(22)	0.012	5.855 (1.467 to 23.368)
*TG* Exon33	TT(33)	R	1
	TC(55)	0.002	7.729 (2.142 to 27.889)
	CC(23)	0.000	15.151 (3.303 to 69.507)
Gender	Male(12)	R	1
	Female(99)	0.829	1.196 (0.234 to 6.120)
Age	-	0.970	1.001 (0.964 to 1.039)

R refers to Reference Group. Data were coded for +49A/G (AA 0, AG 1, GG 2), CT60 A/G(AA 0, AG 1, GG 2), *TG* E33 (TT 0, CT 1, CC 2), gender (Male 0, Female 1) and age (years, continuous variable).

Furthermore, there was significantly high frequency of the G allele in patients with autoimmune hypothyroidism compared with controls (55% vs. 31% respectively; χ^2^ = 12.58, *p* = 0.0004) (Table 1). Both patient and control populations were found to be in Hardy-Weinberg equilibrium for this polymorphism (*p* = 0.2783 and *p* = 0.0913 respectively) (Table 1). This study has 83.01% statistical power for the effect size 0.5 to detect association of +49A/G polymorphism of *CTLA4* at p<0.05 in patients and control population.

### Analysis of association between 3’ UTR *CTLA4* gene CT60A/G polymorphism and susceptibility to autoimmune hypothyroidism

The genotyping of CT60A/G polymorphism revealed a 216 bp undigested product corresponding to G allele and 174 bp and 42 bp digested products corresponding to A allele by PCR-RFLP method. The three genotypes identified by 3.5% agarose gel electrophoresis were: AA homozygous, AG heterozygous and GG homozygous for CT60A/G polymorphism of *CTLA4* gene (S1B Fig).

AG and GG genotypes of 3’UTR CT60 polymorphism of *CTLA4* gene when compared with AA genotype between patients and control by chi-square test-2x2 contingency table showed to increase susceptibility to autoimmune hypothyroidism (*p* = 0.0110 and odds ratio 4.853; *p* = 0.0005 and odds ratio 7.174 respectively) (Table 1). Furthermore logistic regression analysis showed that, when adjusted for age and gender, AG and GG genotype of 3’UTR CT60 polymorphism of *CTLA4* gene increase the risk of autoimmune hypothyroidism by 7.096 fold (95% CI: 1.616 to 31.171, *p* = 0.009) and 5.855 fold (95% CI: 1.467 to 23.368, *p* = 0.012) respectively (Table 2).

Furthermore, there was significantly high frequency of the G allele in patients with autoimmune hypothyroidism compared with controls (77% vs. 51% respectively; χ^2^ = 15.40, *p*<0.0001) (Table 1). Patient population was found to be in Hardy-Weinberg equilibrium for this polymorphism however Control population deviated from the equilibrium (*p* = 0.3008 and *p* = 0.0004 respectively) (Table 1). This study has 83.01% statistical power for the effect size 0.5 to detect association of +49A/G polymorphism of *CTLA4* at p<0.05 in patients and control population.

### Analysis of association between *TG* gene E33 polymorphism and susceptibility to autoimmune hypothyroidism

The genotyping of *TG* gene Exon 33 polymorphism revealed a 375 bp undigested product corresponding to T allele and 208 bp and 167 bp digested products corresponding to C allele by PCR-RFLP method. The three genotypes identified by 3.5% agarose gel electrophoresis were: TT homozygous, TC heterozygous and CC homozygous for E33 polymorphism of *TG* gene (S1C Fig).

TC and CC genotypes of E33 polymorphism of *TG* gene when compared with TT genotype between patients and control by chi-square test-2x2 contingency table showed to increase susceptibility to autoimmune hypothyroidism (*p* = 0.0003 and odds ratio 7.519; *p*<0.0001 and odds ratio 20.54 respectively) (Table 1). Furthermore logistic regression analysis showed that, when adjusted for age and gender, TC and CC genotype of 3’UTR CT60 polymorphism of *CTLA4* gene increase the risk of autoimmune hypothyroidism by 7.729 fold (95% CI: 2.142 to 27.889, *p* = 0.002) and 15.151fold (95% CI: 3.303 to 69.507, *p* = 0.000) respectively (Table 2).

Furthermore, there was significantly high frequency of the C allele in patients with autoimmune hypothyroidism compared with controls (62% vs. 39% respectively; χ^2^ = 22.34, *p*<0.0001) (Table 1). Both patient and control populations were found to be in Hardy-Weinberg equilibrium for this polymorphism (*p* = 0.1083 and *p* = 0.9375 respectively) (Table 1). This study has 83.01% statistical power for the effect size 0.5 to detect association of +49A/G polymorphism of *CTLA4* at p<0.05 in patients and control population.

### Linkage disequilibrium (LD) and haplotype analyses

The LD analysis revealed that the two polymorphisms investigated in the *CTLA4* gene were in moderate LD association (+49A/G: CT60A/G; D’ = 0.64, r^2^ = 0.11). A haplotype evaluation of the two polymorphic sites was performed and the estimated frequencies of the haplotypes differed between autoimmune hypothyroidism patients and controls (global *p*-value <0.0001) (Table 3). However, the GG haplotype was more frequently observed in autoimmune hypothyroidism patients and increased the risk of autoimmune hypothyroidism by 4.688-fold [*p* = <0.0001; odds ratio (OR): 4.688; 95% confidence interval (CI): (2.534~8.670)] (Table 3).

**Table 3:** Distribution of haplotypes frequencies for *CTLA4* gene polymorphisms (+49A/G and CT60A/G) among autoimmune hypothyroidism patients and controls.

	Case (freq) (n = 96)	Control (freq) (n = 120)	Chi^2^	P for association	P (global)	Odds ratio (95% CI)
**A A**	17(0.182)	34(0.287)	3.217	0.072945	<0.0001	0.553 [0.288~1.061]
**A G**	25(0.255)	48(0.396)	4.761	0.029157		0.523 [0.291~0.940]
**G A**	6(0.057)	17(0.138)	3.765	0.052383		0.381 [0.140~1.040]
**G G**	48(0.505)	21(0.179)	25.933	<0.0001		4.688 [2.534~8.670]

## Discussion

The present study showed low iodine intake is not the sole etiological candidate for hypothyroidism disorder in India as approximately half of the hypothyroidism patients included in this study were found to have presence of anti-TPO antibodies (S1 Table). Same trend was observed in the recent study conducted on the prevalence of thyroid diseases in eight cities of India and presence of anti-TPO antibodies was shown to be conclusive for the disease [11]; however that study has not reported prevalence of autoimmune hypothyroidism in particular. No relationship between age of the patient and presence of anti-TPO antibodies was found in present study (data not shown), which is in accordance with the previous study [11].

Furthermore, to evaluate the possible expression dysregulation of *CTLA4* variants, which are important in T regulatory cell’s function, the mRNA levels of fl*CTLA4* and s*CTLA4* genes were measured in patients with autoimmune hypothyroidism and compared with those from controls.

Interestingly, we found significantly decreased mRNA expression of both fl*CTLA4* and s*CTLA4* in autoimmune hypothyroidism patients as compared to controls (Fig 1). We further analyzed whether the polymorphisms examined in this study influenced the expression levels of fl*CTLA4* and s*CTLA4*. The +49AG and +49GG genotypes significantly decreased fl*CTLA4* and s*CTLA4* mRNA expression levels in autoimmune hypothyroidism patients compared to controls; whereas, +49AA genotype did not affect mRNA expression levels (Fig 2A & 2B). It has been reported that +49A/G may influence pattern or level of *CTLA4* expression even if not the function of *CTLA4* protein because G allele is associated with reduced control of T cell proliferation and thus contributes to the pathogenesis of autoimmune hypothyroidism, Grave’s disease and other autoimmune diseases [21].

The 3’ UTR of *CTLA4* gene has also been found to be involved in several autoimmune diseases hence to study the genetic variation of such regions is imperative [24] [25] [36]. Interestingly, we found that 3’ UTR CT60G allele greatly reduced the mRNA expression of both fl*CTLA4* and s*CTLA4* in autoimmune hypothyroidism patients as compared to controls suggesting its crucial role in pathogenesis of autoimmune hypothyroidism whereas, CT60AA and CT60AG genotypes did not affect mRNA expression levels (Fig 2C & 2D). However, previous study did not detect any significant difference of s*CTLA4* and fl*CTLA4* mRNA expression based on the CT60 genotype in patients with autoimmune thyroid disorder compared to healthy individuals [37].

Recently studies on type-1 diabetes have also shown decreased s*CTLA4* levels and suggested that lower s*CTLA4* expression may directly affect the suppressive capacity of regulatory T lymphocytes and thereby modulate disease risk [38]. In contrast, increased serum sCTLA4 levels were detected in other autoimmune diseases such as Graves’ disease [39] and autoimmune thyroid disease [40]. These studies suggest that s*CTLA4* might contribute to the development of autoimmune diseases, probably through inhibiting the B7-flCTLA4 interaction and down-regulation of T cell activation.

Moreover, the haplotype GG (+49G: CT60G) greatly decreased mRNA levels of s*CTLA4* and haplotypes GG (+49G: CT60G) and AG (+49A: CT60G) greatly decreased mRNA levels of fl*CTLA4* in patients as compared to controls (data not shown) revealing the positive correlation of +49G and CT60G in autoimmune hypothyroidism pathogenesis. Moreover, ratio of s*CTLA4* to fl*CTLA4* mRNA expression was not found to be altered in autoimmune hypothyroidism patients compared to controls (Fig 3). Moreover s*CTLA4* to fl*CTLA4* mRNA expression ratio was not affected by +49A/G and CT60A/G polymorphisms (Fig 3).

However, elevated s*CTLA4*/fl*CTLA4* mRNA expression ratio was found in patients with the haplotype GG (+49G: CT60G) as compared to controls (Fig 3) showing strong positive correlation of +49G and CT60G in autoimmune hypothyroidism pathogenesis.

The present study found higher frequency of +49AG, +49GG and CT60GG genotypes among autoimmune hypothyroidism patients and this seems to modulate *CTLA4* mRNA expression (Fig 2A, 2B, 2C & 2D) however, +49AA, CT60AA and CT60AG genotype does not seem to modulate *CTLA4* mRNA expression and hence patients harboring it may have other genetic factors involved in disease pathogenesis supporting the fact that being an autoimmune disease autoimmune hypothyroidism may have varied type of precipitating factors [21].

Also in the present study two polymorphic sites in the *CTLA4* gene i.e., exon 1: +49A/G and in the 3’ UTR region CT60A/G, were found to be associated with autoimmune hypothyroidism susceptibility in Gujarat population, as significant difference for genotype and allele frequency was observed between autoimmune hypothyroidism patients and controls (Tables 1 & 2).

In particular, we found that +49 GG and +49 AG genotypes were more prevalent among autoimmune hypothyroidism patients (Tables 1 & 2). These findings are in accordance with white Caucasian and Japanese population [21, 41, 42]. Conversely, the study on Korean population did not find significant difference in genotype and allele frequency for +49A/G polymorphism in autoimmune hypothyroidism patients [20].

We also found that the presence of CT60GG genotype was more frequent among autoimmune hypothyroidism patients than control and G allele was also found to be associated with disease susceptibility (Tables 1 & 2) and our results are in accordance with Japanese and other populations [29], [43]. Further, the haplotypes AG (+49A: CT60G) and GG (+49G: CT60G) were more frequent in patients as compared to controls (Table 3). Our results along with previous studies suggest that the *CTLA4* gene on chromosome 2q33 is a susceptibility locus for autoimmune hypothyroidism [20], [21], [23], [26], [29].

However, it is clear from our results that some patients with AA genotype of +49A/G and CT60 also suffer from autoimmune hypothyroidism whereas some controls with GG genotype +49A/G and CT60 do not develop disease suggesting role of other precipitating factors for autoimmune hypothyroidism being multifactorial disease [21]. However, age and gender were not found to have crucial role in susceptibility to autoimmune hypothyroidism (Table 2).

Present study also investigated association of *TG* E33 polymorphism with autoimmune hypothyroidism susceptibility. The present study found higher frequency of CC genotype for *TG* E33 in autoimmune hypothyroidism patients compared to controls, suggesting its association with autoimmune hypothyroidism. It has been identified that significant association of *TG* E33 and an exon 10–12 SNP cluster with autoimmune thyroid disorders and also the interaction of *TG* E33 with *HLA-DR3* confers susceptibility to autoimmune thyroid disease [31]. The *TG* E33 polymorphism causes the change from a hydrophobic amino acid tryptophan to a positively charged hydrophilic amino acid arginine and this non-conservative amino acid substitution would be expected to change the structure of *TG* at this region [31].

## Conclusion

Our findings show that the +49A/G and 3’ UTR CT60A/G polymorphisms of the *CTLA4* gene influence both full length and soluble *CTLA4* mRNA expression levels in patients with autoimmune hypothyroidism. This suggests variations at the genetic level, at least in part, could lead to the dysregulation of *CTLA4* expressions in autoimmune hypothyroidism patients and supports the autoimmune pathogenesis of the disease. Therefore, further research on relation between various *CTLA4* polymorphisms, dynamics of *flCTLA4* versus s*CTLA4* expression and their turnover in autoimmune hypothyroidism as well as other autoimmune diseases is needed to clarify the role of *CTLA4* in the regulation of immune response. In addition, *TG* gene may also predispose to autoimmune hypothyroidism by the mechanism of protein structure change due to nonconservative amino acid substitution and thus in turn may change its antigenicity making it more immunogenic and could confer susceptibility to autoimmune hypothyroidism.

## Supporting Information

S1 Fig **PCR-RFLP analysis of *CTLA4* exon 1 +49 A/G and 3’ UTR CT60A/G and *TG* E33 polymorphisms: (A)** PCR-RFLP analysis of *CTLA4* exon 1 +49 A/G polymorphism on 3.5% agarose gel electrophoresis: lanes: 3 & 5 show heterozygous (AG) genotypes; lanes: 2 & 4 show homozygous (AA) genotypes; lane: 1, 6 & 7 show homozygous (GG) genotype; lane M shows 100 bp DNA ladder. **(B)** PCR-RFLP analysis of *CTLA4* 3’ UTR CT60A/G polymorphism on 3.5% agarose gel electrophoresis: lanes: 1 & 2 show heterozygous (AG) genotypes; lanes: 5 shows homozygous (AA) genotypes; lane: 3, 4 & 6 show homozygous (GG) genotype; lane M shows 100 bp DNA ladder. **(C)** PCR-RFLP analysis of *TG* E33 polymorphism on 3.5% agarose gel electrophoresis: lanes: 1, 2 & 6 show heterozygous (TC) genotypes; lanes: 4 & 5 show homozygous (TT) genotypes; lane: 3 & 7 show homozygous (CC) genotype; lane M shows 50 bp DNA ladder.
(TIF)

S1 Table Demographic characteristics of hypothyroidism patients and controls. (DOCX)

S2 Table Primers used for genotyping of *CTLA4* and *TG* SNPs and gene expression analysis. (DOC)
