# Supplementary figures and images for: Association of Cytotoxic T-Lymphocyte Antigen 4 (CTLA4) and Thyroglobulin (TG) Genetic Variants with Autoimmune Hypothyroidism
Source: PLoS One. 2016 Mar 10;11(3):e0149441. doi: 10.1371/journal.pone.0149441 (PMC4786160; doi:10.1371/journal.pone.0149441)

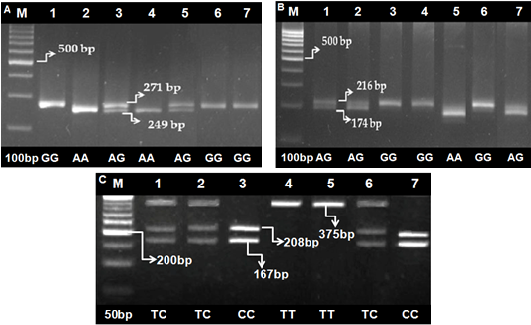

Supplement: S1 Fig — PCR-RFLP analysis of CTLA4 exon 1 +49 A/G and 3’ UTR CT60A/G and TG E33 polymorphisms: (A) PCR-RFLP analysis of CTLA4 exon 1 +49 A/G polymorphism on 3.5% agarose gel electrophoresis: lanes: 3 & 5 show heterozygous (AG) genotypes; lanes: 2 & 4 show homozygous (AA) genotypes; lane: 1, 6 & 7 show homozygous (GG) genotype; lane M shows 100 bp DNA ladder. (B) PCR-RFLP analysis of CTLA4 3’ UTR CT60A/G polymorphism on 3.5% agarose gel electrophoresis: lanes: 1 & 2 show heterozygous (AG) genotypes; lanes: 5 shows homozygous (AA) genotypes; lane: 3, 4 & 6 show homozygous (GG) genotype; lane M shows 100 bp DNA ladder. (C) PCR-RFLP analysis of TG E33 polymorphism on 3.5% agarose gel electrophoresis: lanes: 1, 2 & 6 show heterozygous (TC) genotypes; lanes: 4 & 5 show homozygous (TT) genotypes; lane: 3 & 7 show homozygous (CC) genotype; lane M shows 50 bp DNA ladder. (TIF) [file pone.0149441.s001.tif]
