# Supplementary material for: Association of Cytotoxic T-Lymphocyte Antigen 4 (CTLA4) and Thyroglobulin (TG) Genetic Variants with Autoimmune Hypothyroidism
Source: PLoS One. 2016 Mar 10;11(3):e0149441. doi: 10.1371/journal.pone.0149441 (PMC4786160; doi:10.1371/journal.pone.0149441)
Supplement: S1 Table — (DOCX) [file pone.0149441.s002.docx]

**S1 Table**. **Demographic characteristics of hypothyroidism patients and controls.**

|  | Hypothyroidism Patients (n =84) | | Anti-TPO positive Hypothyroidism Patients (n =50) | | Controls (n=62) | |
| --- | --- | --- | --- | --- | --- | --- |
|  | Female (78) | Male(6) | Female (44) | Male (6) | Female (55) | Male (7) |
| Average age (years, Mean age ± SD) | 43.25 ± 15.16 | 64 ± 9.17 | 43.40 ± 14.01 | 64 ± 9.17 | 41.27±15.27 | 58.33 ± 2.08 |
| Onset Age (years, Mean age ± SD) | 38.72 ± 13.86 | 61.33 ± 9.03 | 38.5 ± 12.25 | 61.33 ± 9.03 | NA | NA |
| Duration of disease (years, Mean age ± SD) | 4.53 ± 3.71 | 2.67 ± 0.67 | 4.91 ± 3.98 | 2.67 ± 0.67 | NA | NA |
