# Supplementary material for: Association of Cytotoxic T-Lymphocyte Antigen 4 (CTLA4) and Thyroglobulin (TG) Genetic Variants with Autoimmune Hypothyroidism
Source: PLoS One. 2016 Mar 10;11(3):e0149441. doi: 10.1371/journal.pone.0149441 (PMC4786160; doi:10.1371/journal.pone.0149441)
Supplement: S2 Table — (DOC) [file pone.0149441.s003.doc]

**S2 Table. Primers used for genotyping of *CTLA4* and *TG*** SNPs and gene expression analysis.

| **SNP/Gene** | **Primers** | **Amplicon**  **size (bp)** | **Annealing Temp (^o^C)** | **Restriction enzyme and product size** |
| --- | --- | --- | --- | --- |
| **(rs231775)** *CTLA4* +49A/G | F 5’-AAGGCTCAGCTGAACCTGGT -3’  R 5’-CTTTGCCTTATTTGCTGCCGC-3’ | 271bp | 60 **^o^C** | *BstEII*  (249 & 22 bp) |
| **(rs3087243)**  *CTLA4* CT60A/G | F 5’-CACCACTATTTGGGATATACC-3’  R 5’-AGCTCTATATTTCAGGAAGGC-3’ | 216bp | 60 **^o^C** | *NcoI*  (174 & 42 bp) |
| *TG* E33 | F: 5’-ATATTGACCAAAGCACCCCC-3’  R: 5’-ATTAGCCAGTTGCCCTCTCC-3’ | 375bp | 60 **^o^C** | Hpy99I (208 & 167 bp) |
| *flCTLA4* gene expression | F: 5’-TATGTAATTGATCCAGAACCGTGC-3’  R: 5’-TAGCATTTTGCTCAAAGAAACAG-3’ | 123bp | 63 **^o^C** | _ |
| *sCTLA4* gene expression | F: 5’-GAACCCAGATTTATGTAATTGCTAAG-3’  R: 5’-CACATTCTGGCTCTGTTGGG-3’ | 88bp | 63 **^o^C** | _ |
| *GAPDH* gene expression | F: 5’-ATCCCATCACCATCTTCCAGGA-3’  R: 5’-CAAATGAGCCCCAGCCTTCT-3’ | 122bp | 63 **^o^C** | _ |
